# Anterior mediastinal paraganglioma: A case for preoperative embolization

**DOI:** 10.1186/1477-7819-10-134

**Published:** 2012-07-03

**Authors:** Murtaza Shakir, Geoff Blossom, John Lippert

**Affiliations:** 1 Department of Surgery, Riverside Methodist Hospital, 3535 Olentangy River Road, Columbus, OH, 43214-3998, USA; 2 Department of Radiology, Division of Vascular and Interventional Radiology, Riverside Methodist Hospital, 3525 Olentangy River Road, Columbus, OH, 43214, USA

**Keywords:** Paraganglioma, Surgical oncology, CT-guided biopsy

## Abstract

**Background:**

Paraganglioma is a rare but highly vascular tumor of the anterior mediastinum. Surgical resection is a challenge owing to the close proximity to vital structures including the heart, trachea and great vessels. Preoperative embolization has been reported once to facilitate surgical treatment.

**Case presentation:**

We report a case of anterior mediastinal paraganglioma that was embolized preoperatively, and was resected without the need for cardiopulmonary bypass and without major bleeding complications.

**Conclusion:**

We make a case to further the role of preoperative embolization in the treatment of mediastinal paragangliomas.

## Background

Pheochromocytomas located outside of the adrenal glands are referred to as paragangliomas [1]. Complete surgical resection, although challenging, renders long-term survival rates up to 84%, making it the treatment modality of choice [2]. These tumors are highly vascular and rarely found in the anterior mediastinum. We present a case of anterior mediastinal paraganglioma that was embolized preoperatively. This, we believe, led to significantly lower blood loss, allowing complete resection without the need for cardiopulmonary bypass.

## Case presentation

**Figure 1:**
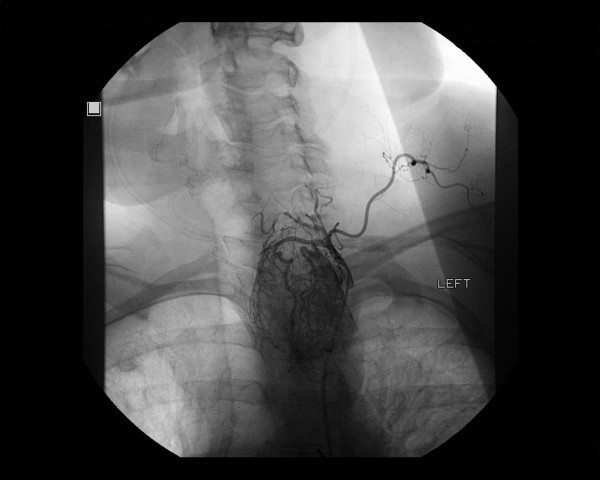
Angiographic image of the mediastinal mass.

A 50-year-old patient had been having recurrent headaches, and right upper and lower extremity weakness, which was worked up. Thoracic surgery was consulted after finding a 4.3 × 5.7 × 8.7-cm mass in the antero-superior mediastinum; see Figure 1. It was located between the origins of the left subclavian and left common carotid arteries, displacing without compressing both vessels. It extended as cephalad as the left lobe of the thyroid gland. It was causing compression of the left brachiocephalic and left internal jugular veins. No other masses or cervical adenopathy was noted. Urine levels of epinephrine, norepinephrine, VMA, metanephrine and normetanephrine were within normal limits.

A CT-guided biopsy of the mediastinal mass was performed using a 19-gauge needle. No complications were encountered. Pathologic examination revealed findings typical of a paraganglioma. Nests of cells with uniform bland nuclei and abundant pink granular cytoplasm in an organoid pattern were seen. Chromogranin and S-100 staining of the sustentacular cells was strongly positive.

An interventional radiology consultation was obtained for an angiogram and possible embolization before undertaking surgical resection of the tumor. This revealed the proximal great vessels were widely patent without significant luminal irregularity or stenosis. However, there was a mild mass effect with splaying of proximal left common carotid and left subclavian arteries by the underlying mass. Two main branches of the left thyrocervical trunk were supplying the large hypervascular mass in the left cervical region extending to the aortic arch. The tumor demonstrated marked hypervascularity with multiple dilated and ectatic outflow veins. There was another small arterial branch arising from left inferior thyroidal artery. Selective embolization of the two main feeding vessels from the left thyrocervical trunk was performed. There was minimal vascularity of the tumor following embolotherapy.

The patient was taken to the operating room 24 h later for definitive resection using median sternotomy. The tumor originated between the left subclavian and left common carotid arteries. It was sharply dissected away from the mediastinal pleura, but the pleural cavity was entered. It was dissected from the pericardium and great vessels with relative ease without major blood loss. Although we were ready for the possibility, cardiopulmonary bypass was not needed. The tumor extended along the left common carotid and internal jugular veins into the neck, with an anatomic plane allowing safe dissection. Final pathology revealed an encapsulated paraganglioma. Hemostasis was adequate, and there no major intraoperative or postoperative bleeding problems. Our patient had an uneventful postoperative recovery and continues to do well 6 months postoperatively.

## Conclusions

Pheochromocytomas are neuroendocrine tumors originating from enterochromaffin cells in the adrenal medulla or sympathetic ganglia that secrete catecholamines [1]. Paragangliomas are extra-adrenal pheochromocytomas, rarely located in the mediastinum. If these tumors are functional, they can classically present with sustained arterial hypertension, headaches and diaphoresis [3]. However, these are rare, representing only 2% of all catecholamine-secreting tumors. Most mediastinal paragangliomas are non-functional. These are mostly asymptomatic but can present with compressive local symptoms. Detection of plasma metanephrines is highly sensitive for detection of functional tumors [4].

The incidence of malignancy in extra-adrenal paragangliomas has been reported between 21% and 76% [5]. After establishing the diagnosis of paraganglioma, imaging studies allow anatomic localization of the tumor. We used a CT-guided biopsy to confirm the diagnosis before operative planning. These tumors are not sensitive to chemotherapy or radiation, and complete surgical resection provides the only chance for cure. This allows long-term (10-year) survival in 84% of patients with complete and 50% with incomplete resection [2].

Our case was that of a biopsy proven anterior mediastinal paraganglioma. The surgery of anterior and middle mediastinal paragangliomas is challenging because of their close proximity to the central airways, heart and great vessels, making complete resection difficult. Preoperative embolization is an established technique to reduce operative blood loss in head and neck tumors, and has been described for mediastinal paragangliomas as well [6]. The hypervascular nature of these tumors is well known, and substantial hemorrhage has been reported during resection [6]. The tumor vascularity was remarkably reduced by preoperative embolization, and this facilitated a safe resection without need of cardiopulmonary bypass. We make a case to further the role of preoperative embolization in the treatment of mediastinal paragangliomas.

## Consent

Written informed consent was obtained from the patient for publication of this case report and any accompanying images. A copy of the written consent is available for review by the Editor-in-Chief of this journal.

## Competing interests

None of the authors have any personal or financial competing interests to report.

## Author contributions

All authors made substantive intellectual contributions this case report, including acquisition of data, interpretation of the case report and drafting/editing of the manuscript, and have given final approval of the version to be published.

## Authors’ information

M.S. is a 5th year general surgery resident.
